# Whole-genome sequencing reveals the genomic characterization and evolution of carbapenem-resistant *Klebsiella pneumoniae* in Gansu, China

**DOI:** 10.3389/fcimb.2026.1749978

**Published:** 2026-04-02

**Authors:** Juanjuan Hou, Juan Liu, Mingliang Guo, Juan Li, Chen Qin, Guoping Zhang

**Affiliations:** 1 Precision Medicine Testing Center, The People’s Hospital of Qingyang City/Qingyang Hospital of the Second Hospital of Lanzhou University, Qingyang, China; 2 Department of Infectious Disease, The People’s Hospital of Qingyang City/Qingyang Hospital of the Second Hospital of Lanzhou University, Qingyang, China; 3 Department of Clinical Laboratory, Lanzhou Second People’s Hospital, Lanzhou, China; 4 Department of Clinical Laboratory, The People’s Hospital of Qingyang City/Qingyang Hospital of the Second Hospital of Lanzhou University, Qingyang, China; 5 Department of Clinical Laboratory, Qingyang Second People’s Hospital, Qingyang, China; 6 Department of Clinical Laboratory, Gansu Provincial Maternity and Childcare Hospital (Gansu Province Central Hospital), Lanzhou, China

**Keywords:** capsular serotype, carbapenem-resistant *Klebsiella pneumoniae*, multilocus sequence typing, virulence genes, whole-genome sequencing

## Abstract

**Introduction:**

*Klebsiella pneumoniae* has emerged as a major opportunistic pathogen, with rising antimicrobial resistance representing a critical public health concern. This study investigated the genomic characteristics, transmission dynamics, and evolutionary relationships of CRKP isolates from Gansu Province, China, to inform regional prevention strategies.

**Methods:**

Thirty CRKP isolates collected from two tertiary hospitals (2023-2025) were tested for susceptibility to 15 antibiotics and subjected to whole-genome sequencing. Genome assembly and annotation employed standard pipelines, followed by multilocus sequence typing, capsular typing, phylogenetic reconstruction, pan-genome, and mobile genetic element analyses. Comparative genomic and statistical analyses were performed with publicly available data.

**Results:**

Most isolates originated from sputum (40%) and blood (23.3%) and were resistant to over 90% of β-lactam and carbapenem agents. Their genomes (5.2-5.8 Mb) contained on average 5,596 coding sequences. ST11 predominated (88.46%), mainly associated with KL25 (64.29%) and KL64 (17.86%) capsule types. The plasmid-borne *blaKPC-2* gene occurred in 79.31% of isolates, while virulence factors (rmpA, rmpA2, iuc) were enriched in ST11-KL25/KL64 lineages (p<0.05). Phylogenomic analysis showed limited genetic diversity (median SNP = 103.5), consistent with clonal spread and potential hospital transmission. Pan-genome comparisons revealed that ST11-KL25 and ST11-KL64 strains shared a conserved core genome but overlapped with non-ST11 strains by only 6.28%, highlighting substantial genomic flexibility across capsule types.

**Conclusions:**

The ST11-KL25 CRKP lineage shows enhanced adaptation and may become or is becoming the dominant clone in Gansu. These results highlight the necessity for continuous, longitudinal genomic surveillance to curb the spread of highly resistant *K. pneumoniae*.

## 1 Introduction

*Klebsiella pneumoniae* is a common opportunistic pathogen implicated in severe infections such as pneumonia, intra-abdominal, urinary tract, and bloodstream infections. Its high morbidity and mortality rates pose major threats to global public health (Reid et al., 2019; Martin and Bachman, 2018). In recent years, the extensive use of broad-spectrum antibiotics has contributed to an escalating antimicrobial resistance burden, particularly the emergence and spread of carbapenem-resistant *K. pneumoniae* (CRKP), which has drastically reduced effective therapeutic options (Micozzi et al., 2017).

The primary mechanism underpinning carbapenem resistance in *K. pneumoniae* involves carbapenemase production. *K. pneumoniae* carbapenemase (KPC) represents the most prevalent class A enzyme. Class B carbapenemases include metallo-β-lactamases such as IMP, VIM, and NDM (New Delhi metallo-β-lactamase), while class D encompasses OXA-type enzymes initially identified in *Acinetobacter* species, these resistance determinants are commonly located on mobile genetic elements, including plasmids and transposons, facilitating horizontal gene transfer across bacterial strains and promoting rapid and widespread CRKP dissemination (Chen et al., 2014; Campos-Madueno et al., 2022). A multicenter study in China that analyzed 1,801 carbapenem-resistant *Enterobacteriaceae* (CRE) isolates collected from 65 hospitals (2012–2016) reported that *K. pneumoniae* was the predominant species, accounting for 91% of isolates, with KPC-producing strains comprising 77% of all CRKP (Lynch et al., 2021). Moreover, surveillance data from the China Antimicrobial Resistance Surveillance System revealed a dramatic increase in meropenem resistance among *K. pneumoniae*, from 2.9% in 2005 to 30.0% in 2023 (Hu et al., 2024).

Whole-genome sequencing (WGS) can be used to comprehensively elucidate CRKP genomic characteristics, transmission dynamics, and evolutionary relationships, thereby providing critical insights for CRKP clinical management and infection control (Jiang et al., 2025). In this study, 30 clinical CRKP isolates were collected from two tertiary hospitals in Gansu Province (2023-2025). Antimicrobial susceptibility testing and WGS were conducted to characterize resistance profiles, virulence factors, and genetic backgrounds. Comparative analyses with publicly available genomic data were also performed. We sought to clarify local transmission patterns and evolutionary trajectories of CRKP antimicrobial resistance, thereby contributing to evidence-based strategies for regional prevention, control, and clinical treatment.

## 2 Materials and methods

### 2.1 Strain collection and identification

A total of 30 non-duplicate CRKP isolates were consecutively collected from clinical specimens at Gansu Provincial Central Hospital and Qingyang People’s Hospital between October 2023 and February 2025. CRKP is defined as *Klebsiella pneumoniae* exhibiting resistance to at least one carbapenem agent, for example, the minimum inhibitory concentration (MIC) of ertapenem is ≥2µg/mL, or the MICs of imipenem, meropenem or doripenem are ≥4µg/mL (Guo et al., 2024). Patient demographic and clinical data, including age, sex, and diagnosis, were recorded. Species identification was performed using matrix-assisted laser desorption/ionization time-of-flight mass spectrometry (BioMérieux, France). Antimicrobial susceptibility testing was conducted using the VITEK 2 automated microbial identification system (BioMérieux, France). Minimum inhibitory concentrations were interpreted according to Clinical and Laboratory Standards Institute M100 guidelines, and tigecycline susceptibility was determined following recommendations from the European Committee on Antimicrobial Susceptibility Testing. *Escherichia coli* ATCC 25922 and *K. pneumoniae* ATCC 700603 (American Type Culture Collection, Manassas, VA, USA) were used as quality control strains. Carbapenemase production was detected using a commercial carbapenemase detection kit (Tianjin Yirui Biotechnology Co., Ltd.) (see **Table 1** for results). The study protocol was approved by the Institutional Medical Ethics Committee (approval number: 202202-04).

**Table 1:** Strain characteristics and collection information.

SampleID	Department	Clinical diagnosis	Age	Gender	Sample source	Carbapenemase phenotype
s1	Rehabilitation Department	High paraplegia	23	male	Mid-stream urine	KPC
s2	Neurology Department	Cerebral infarction	78	male	Sputum	KPC
s3	Intensive Care Unit	Femoral fracture	53	male	Mid-stream urine	KPC
s4	Rehabilitation Department	Craniocerebral injury	64	female	Sputum	KPC
s5	Emergency Intensive Care Unit	Septicopyemia	71	female	Blood	KPC
s6	Rehabilitation Department	Dyskinesia	55	male	Sputum	KPC
s7	Neurosurgery Department	Hemorrhage of brain stem	55	male	Sputum	KPC
s8	Intensive Care Unit	Severe pneumonia	75	male	Sputum	KPC
s9	Rehabilitation Department	Paraplegia	40	male	Mid-stream urine	KPC
s10	Nedical Oncology	Lung cancer	38	female	Intravenous Tube	KPC
s11	Neurology Department	Shock	59	male	Blood	KPC
s12	Rehabilitation Department	Dyskinesia	76	male	Sputum	KPC
s13	Department of Radiotherapy	Lung cancer	42	male	Sputum	KPC
s14	Neurosurgery Department	Intracranial space-occupying lesion	54	male	Sputum	KPC
s15	Neurosurgery Department	Intracranial space-occupying lesion	54	male	Sputum	KPC
s16	Rehabilitation Department	Dyskinesia	58	male	Sputum	KPC
s17	Rehabilitation Department	Dyskinesia	53	male	Mid-stream urine	KPC
s18	Rehabilitation Department	Cerebral hemorrhage	55	female	Mid-stream urine	KPC
s19	Rehabilitation Department	Encephalitis	43	female	Blood	KPC
s20	Emergency Intensive Care Unit	Cerebral infarction	66	male	Mid-stream urine	KPC
s21	Neurosurgery Department	Subarachnoid hemorrhage	61	male	Intravenous Tube	KPC
s22	Emergency Intensive Care Unit	Spinal cord injury	16	male	BALF	KPC
s23	Rehabilitation Department	Grade 3 hypertension	59	male	Sputum	KPC
s24	Rehabilitation Department	Brachial plexus nerve damage	61	male	Mid-stream urine	KPC
s25	Emergency Intensive Care Unit	Septic Shock	80	male	Blood	KPC
s26	Neurosurgery Department	Cerebral hemorrhage	44	female	Blood	NDM
s27	Intensive Care Unit	Severe pneumonia	56	female	Sputum	NDM
s28	Intensive Care Unit	Septicopyemia	49	female	Blood	KPC+OXA23
s29	Neurology Department	Severe pneumonia	65	male	BALF	KPC+NDM
s30	Neurosurgery Department	Cerebral hemorrhage	48	male	Blood	OXA23

### 2.2 WGS

Genomic DNA was extracted from isolates using a commercial bacterial DNA extraction kit(Beijing TianGen Biochemical Technology Co., Ltd.). DNA purity and concentrations were evaluated spectrophotometrically to ensure high-quality templates for sequencing. WGS was performed on the Illumina x plus platform(Illumina, Inc., San Diego, CA, USA), generating 150 bp paired-end reads with an average depth exceeding 100× to ensure comprehensive genomic coverage. Sequencing data were deposited in the National Center for Biotechnology Information (NCBI) database under BioProject accession number https://www.ncbi.nlm.nih.gov/bioproject/PRJNA1331570.

### 2.3 Genome assembly and annotation

Raw sequencing reads were filtered to remove low-quality bases and adapters, yielding high-quality clean reads (**Table 2**). Genome assembly was conducted using multiple assemblers-SOAPdenovo (version 2.04) (Xie et al., 2014), SPAdes (Prjibelski et al., 2020), and ABySS (Simpson et al., 2009)-followed by assembly integration using CISA software. Resulting assemblies were refined by gap filling and local optimization in GapCloser (version 1.12) (Xu et al., 2020). Contigs shorter than 500 bp were excluded from further analysis, and potentially contaminated samples underwent additional decontamination. Quality assessment, statistical characterization, and gene predictions were also performed. Scaffold-level assembly statistics are shown (**Table 2**). Repetitive elements were identified using RepeatMasker (version open-4.0.5) (Tarailo-Graovac and Chen, 2009) and tandem repeats were detected using Tandem Repeats Finder (TRF, version 4.07b) (Benson, 1999). Protein-coding genes were predicted in GeneMarkS (version 4.17) (Besemer et al., 2001). Non-coding RNAs were annotated using tRNAscan-SE (version 1.3.1) (Chan and Lowe, 2019) for transfer RNAs (tRNAs), rRNAmmer (version 1.2) (Lagesen et al., 2007) for ribosomal RNAs (rRNAs), and Infernal’s cmsearch (version 1.1rc4) (Cui et al., 2016) for small RNAs (sRNAs).

**Table 2:** Summary of whole-genome sequencing metrics.

SampleID	Genome size	N50	GC%	Gene number	Gene average length	tRNA
s1	5868657	193946	56.98	5759	882	78
s2	5663997	374146	57.08	5424	909	81
s3	5864834	152220	57.01	5759	885	76
s4	5435406	474613	57.25	5178	920	76
s5	5915130	131216	56.87	5812	880	78
s6	5866235	147406	56.97	5776	881	79
s7	5885265	147299	56.97	5788	881	79
s8	5830422	152897	57.00	5738	883	76
s9	5866219	146167	56.98	5767	881	78
s10	5873098	147130	56.98	5764	881	78
s11	5896408	146322	56.98	5793	882	78
s12	5898859	130707	56.97	5804	883	80
s13	5909941	147565	56.98	5804	883	79
s14	5904700	175015	56.95	5809	880	80
s15	5897661	169187	56.92	5788	877	78
s16	5873501	146016	57.02	5764	884	78
s17	5895486	148036	57.02	5803	883	78
s18	5867564	130582	56.99	5783	880	78
s19	5936029	146604	56.94	5845	880	76
s20	5834302	152284	57.04	5725	887	76
s21	5868939	152908	57.02	5765	885	78
s22	5864665	130580	57.02	5742	887	77
s23	5843817	193631	57.05	5743	885	77
s24	5886052	152791	57.00	5786	885	75
s25	5858606	152149	57.03	5742	887	77
s26	5138856	134606	50.6	4941	900	82
s27	5762565	375374	56.58	5511	903	83
s29	6582597	262251	55.34	6332	904	80

### 2.4 Functional gene annotation

Gene functions were predicted using homology-based searches against multiple functional databases, including Gene Ontology (GO), Kyoto Encyclopedia of Genes and Genomes (KEGG), Clusters of Orthologous Groups of proteins (http://www.ncbi.nlm.nih.gov/COG/), Non-Redundant Protein, Transporter Classification, Swiss-Prot (http://www.ebi.ac.uk/uniprot/), and Carbohydrate-Active enZYmes Databases. The functional annotation workflow comprised the following steps: (1) protein sequences were compared with each database using BLASTp, with an E-value threshold ≤ 1e-5; and (2) resulting alignments were filtered to retain the best hit for each query sequence, which was based on the highest alignment score and default thresholds of ≥ 40% sequence identity and ≥ 40% coverage. These thresholds were selected based on established practices in protein homology searching, balancing sensitivity for detecting distant homologs with specificity for minimizing false-positive annotations (Altschul et al., 1997). Secondary metabolite gene clusters were identified using antiSMASH (version 2.0.2). Type III secretion system-related proteins in Gram-negative bacteria were annotated using database matches. Virulence factors and antibiotic resistance genes were further characterized using pathogen-specific databases, including Virulence Factor (Liu et al., 2022), Antibiotic Resistance Genes (Alcock et al., 2023), and Comprehensive Antibiotic Resistance Databases (Alcock et al., 2023).

### 2.5 Capsular genotyping and multilocus sequence typing

Capsular genotypes were determined by the sequence analysis of the K-locus (KL) region using the Kaptive tool (Lam et al., 2022), which identified capsular types (KL types) and evaluated KL completeness and reliability. Typing accuracy was validated using wzi gene–based sequence typing. Comprehensive genomic characterization was further performed using Kleborate (Wyres et al., 2016), which simultaneously detected MLST, antimicrobial resistance genes, and virulence factors, thereby providing a robust molecular profile for each strain. This analysis enabled the rapid and accurate identification of sequence types (STs), capsular types, resistance gene repertoires, and hypervirulence-associated markers for detailed risk assessments. MLST analysis was based on seven housekeeping genes (*gapA, infB, mdh, pgi, phoE, rpoB*, and *tonB*), following the standard *K. pneumoniae* MLST scheme (Ibarz Pavón and Maiden, 2009). Allelic profiles were compared with reference sequences in the MLST database, and each isolate was assigned an ST accordingly. Clonal complexes were subsequently determined based on allelic relatedness.

### 2.6 Phylogenetic analysis

The reference genome K. pneumoniae strain KB (GenBank accession no. CP029384.2) was selected for alignments based on the following criteria: (1) it is a complete, closed genome with high-quality annotation; (2) it belongs to the ST11 lineage, the predominant sequence type in this study; (3) it originated from Sichuan Province, China, representing one of the earliest reported ST11-CRKP strains (2018); and (4) it has been used as a reference in several recent CRKP genomic studies. Seventy-two K. pneumoniae strains previously isolated from diverse regions of China (BioProject PRJNA1061342) were included for comparative analysis. These strains were collected between 2017 and 2023 from multiple provinces, including Shanxi, Inner Mongolia, Ningxia, and Henan, representing diverse clinical sources and geographical origins (Wang et al., 2024). Core-genome single nucleotide polymorphisms (SNPs) were identified using Snippy (https://github.com/tseemann/snippy) and a maximum likelihood phylogenetic tree was reconstructed using FastTree. The resulting phylogeny was visualized using FigTree. To further contextualize evolutionary relationships, additional *K. pneumoniae* genomes representing global genetic diversity were retrieved from GenBank and incorporated into analyses. Pan-genome analysis was performed using Roary (Sitto and Battistuzzi, 2020) to define a shared core gene set among isolates, followed by maximum likelihood phylogenetic tree construction in FastTree (Price et al., 2010). Branch support was evaluated using 1,000 bootstrap replicates. To quantify pairwise genomic similarity, average nucleotide identity (ANI) values were calculated using FastANI.

### 2.7 Mobile genetic element analysis

Putative plasmid-derived contigs were identified using PlasFlow (Krawczyk et al., 2018) and integrative conjugative elements detected using IntegronFinder (Néron et al., 2022) under default parameters. Genomic islands were predicted using IslandPath-DIOMB (Bertelli and Brinkman, 2018), which identified horizontally transferred regions based on dinucleotide bias and mobility-related genes such as transposases and integrases. Prophage regions were predicted using PHASTER (Arndt et al., 2016) and both complete and incomplete prophage elements were enumerated for each isolate. Clustered regularly interspaced short palindromic repeat (CRISPR) arrays were identified with CRISPRdigger (Ge et al., 2016), an optimized search tool that detects short palindromic repeat structures in bacterial genomes.

### 2.8 Data integration and statistical analysis

Data processing and statistical analyses were performed in R software. Multiple regression analyses were conducted to evaluate the influence of co-occurring antimicrobial resistance genes and virulence factors. Associations between phenotypic traits and genotypic characteristics were assessed using chi-square or Fisher’s exact tests, as appropriate. A p-value<0.05 indicated statistical significance. Principal component analysis and hierarchical clustering were applied to identify genetic subpopulations, which were compared with clinical variables to explore potential epidemiological and phenotypic correlations.

## 3 Results

### 3.1 Clinical characteristics of CRKP isolates

**Figure 1:**
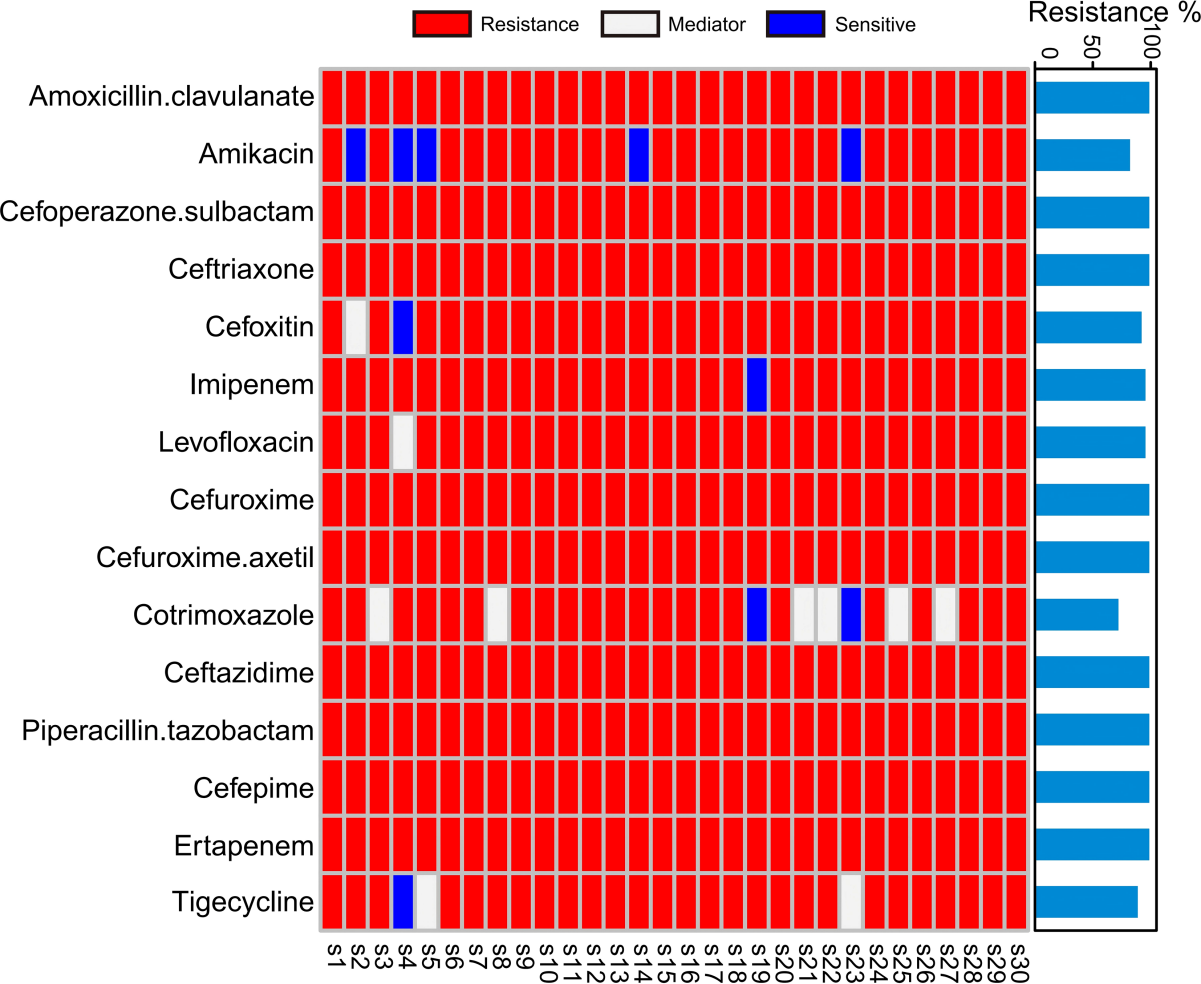
Distribution of antibiotic resistance profiles among clinical isolates. The heatmap displays the antimicrobial susceptibility patterns of 30 CRKP strains to 15 antibiotics, where red represents resistance, gray indicates intermediate resistance, and blue denotes susceptibility.

Thirty clinical CRKP isolates came from blood (n=7), sputum (n=12), vascular catheters (n=2), urine (n=7), and bronchoalveolar lavage fluid (n=2). Patient ages ranged from 16 to 80 years, comprising 22 males and 8 females. Most patients (80%) presented with underlying pulmonary or neurological diseases, such as pneumonia, lung cancer, or cerebral hemorrhage. Antimicrobial susceptibility testing was conducted against 15 antibiotics (**Figure 1**). All isolates exhibited resistance to amoxicillin/clavulanic acid, cefoperazone/sulbactam, ceftriaxone, cefuroxime, cefuroxime axetil, ceftazidime, piperacillin/tazobactam, cefepime, and ertapenem. Amikacin resistance was observed in 83.33% of isolates, whereas 16.67% remained susceptible. Resistance percentages to cefoxitin, imipenem, levofloxacin, cotrimoxazole, and tigecycline were 93.33%, 96.67%, 96.67%, 73.33%, and 90.00%, respectively, with corresponding susceptibility percentages of 3.33%, 3.33%, 0.00%, 6.67%, and 3.33%.

### 3.2 WGS and genomic features

Quality assessments showed that two isolates (s28 and s30) had aberrantly low GC content (38.94% and 38.76%, respectively), mapping rates below 80%, and average nucleotide identity (ANI) values below the 95% species demarcation threshold, so they were excluded due to suspected contamination in the following analysis. Genome sizes among the 28 isolates ranged from 5.13 Mb to 6.58 Mb, with an average GC content of 56.69%. Assembly generated an average of 42 contigs or scaffolds per strain, with an average N50 value of 182630 bp. Genome annotation identified approximately 5723 protein-coding genes per isolate, along with 75–83 tRNA genes and 25 rRNA operons. Non-coding RNA analysis revealed an average of eight sRNAs per strain, potentially involved in antimicrobial resistance and virulence regulation. All genomes achieved a sequencing depth exceeding 100×, ensuring high-quality data for subsequent analyses. Detailed assembly and annotation statistics are shown (**Table 2**).

**Figure 2:**
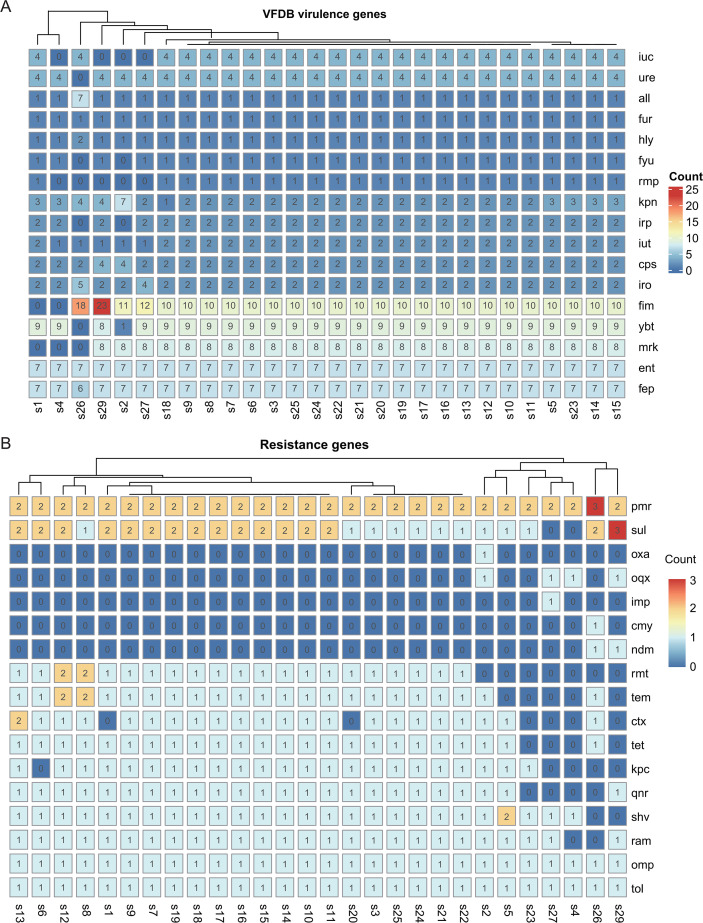
Virulence factor and antimicrobial resistance gene profiles in clinical isolates of carbapenem-resistant Klebsiella pneumoniae (CRKP). **(A)** Distribution of major virulence factors across strains. **(B)** Distribution patterns of antimicrobial resistance genes across strains.

Functional classification indicated that genes related to carbohydrate metabolism (15.7%), amino acid metabolism (13.1%), and membrane transport (11.5%) represented the most abundant categories. GO annotation showed that catalytic activity (42.3%) and binding (39.7%) were the dominant molecular function terms, while metabolic processes (28.7%) and cellular processes (25.9%) were most prevalent among biological processes. KEGG pathway enrichment analysis further revealed that carbohydrate metabolism, amino acid metabolism, and antibiotic biosynthesis pathways were highly represented. Genome annotation identified an average of 11 glycoside hydrolase genes (**Supplementary Table 1**) and 68 glycosyltransferase genes (**Supplementary Table 1**) per strain, which were likely involved in capsular polysaccharide (CPS) biosynthesis and biofilm formation. Virulence factor analysis demonstrated that all isolates harbored the *cps* gene cluster, iron acquisition systems (*fur*, *iro*), adhesion-associated genes (*all*), and hemolysin-related genes (*hly*) (**Figure 2A**). Resistance-associated genes primarily included those encoding KPC-type carbapenemases (kpc), extended-spectrum β-lactamases (ESBLs; ctx, shv), outer membrane protein variants due to gene mutations (omp), and AcrAB-TolC efflux system components (tol) (**Figure 2B**). In addition, 93.43% of isolates contained CRISPR-Cas systems, with an average of 11.7 CRISPR arrays per genome (**Supplementary Table 2**), suggesting possible defense roles against bacteriophage infection. Mobile genetic element analysis revealed an average of 6.6 insertion sequences and 1.5 integrase genes per strain, indicating a capacity for horizontal antimicrobial resistance gene transfer. Prophage prediction identified an average of 5.4 complete and 9 incomplete prophage elements per genome (**Supplementary Table 3**), which potentially facilitated horizontal gene transfer and contributed to virulence and resistance determinant dissemination. Additionally, an average of 14.43 genomic islands were detected per strain, each containing approximately 12.16 intact genes (**Supplementary Table 4**).

### 3.3 Multilocus sequence typing and capsular genotyping

The integration of MLST and Kleborate analyses enabled ST assignment for 26/28 isolates, which were classified into four distinct STs (**Table 3**). The unsuccessful typing of isolates s26 and s29 was attributed to technical issues: incomplete assembly and contig fragmentation of the K-locus region hindered accurate capsular genotype assignment. ST11 was the predominant type, accounting for 88.46% (23 isolates), followed by ST307, ST1393, and ST45-1LV, each represented by a single isolate (3.85%). Capsular genotyping based on the KL was successful for 28 isolates, revealing seven distinct capsular types. KL25 was the most prevalent (18 isolates, 64.29%), followed by KL64 (5 isolates, 17.86%), whereas the remaining isolates were assigned to KL8, KL62, KL145, KL101, and KL102 (**Table 3**).

**Table 3:** Sequence typing and capsular genotype distribution among CRKP isolates.

SampleID	KL	ST	gapA	infB	mdh	pgi	phoE	rpoB	tonB
s1	KL64	ST11	3	3	1	1	1	1	4
s2	KL102	ST307	4	1	2	52	1	1	7
s3	KL25	ST11	3	3	1	1	1	1	4
s4	KL62	ST45-1LV	2	1	1	6	7	–	12
s5	KL64	ST11	3	3	1	1	1	1	4
s6	KL25	ST11	3	3	1	1	1	1	4
s7	KL25	ST11	3	3	1	1	1	1	4
s8	KL25	ST11	3	3	1	1	1	1	4
s9	KL25	ST11	3	3	1	1	1	1	4
s10	KL25	ST11	3	3	1	1	1	1	4
s11	KL25	ST11	3	3	1	1	1	1	4
s12	KL25	ST11	3	3	1	1	1	1	4
s13	KL25	ST11	3	3	1	1	1	1	4
s14	KL64	ST11	3	3	1	1	1	1	4
s15	KL64	ST11	3	3	1	1	1	1	4
s16	KL25	ST11	3	3	1	1	1	1	4
s17	KL25	ST11	3	3	1	1	1	1	4
s18	KL25	ST11	3	3	1	1	1	1	4
s19	KL25	ST11	3	3	1	1	1	1	4
s20	KL25	ST11	3	3	1	1	1	1	4
s21	KL25	ST11	3	3	1	1	1	1	4
s22	KL25	ST11	3	3	1	1	1	1	4
s23	KL64	ST11	3	3	1	1	1	1	4
s24	KL25	ST11	3	3	1	1	1	1	4
s25	KL25	ST11	3	3	1	1	1	1	4
s26	KL101	--							
s27	KL8	ST1393	2	1	37	2	3	1	19
s29	--	--							

### 3.4 Virulence and antimicrobial resistance gene profiles

Identified virulence genes included those associated with capsule synthesis (*rmpA, rmpA2, magA, wzi, cps)*, iron acquisition (*iuc, iut, iro, ybt, kfu, fep, irp*), adhesion and invasion (*fim, mrk, allABC, cf29A, kpn*), toxins and invasins (*clb, hly, cnf*), and iron regulation (*fur, hmu*), as annotated using the Virulence Factor Database. All isolates carried the core virulence determinants cps and the iro-mediated iron acquisition system (**Figure 2A**).

Comparative analysis revealed that ST11-KL25 and ST11-KL64 isolates possessed significantly more virulence-associated genes than other ST-KL types (63.39 *vs*. 57.8; p< 0.05). Both lineages carried rmpA/rmpA2 and the iuc locus encoding aerobactin, a defining feature of hypervirulent *K. pneumoniae* (hvKP). In addition, ST11-KL25 isolates harbored the ybt siderophore system and *irp1/irp2* genes, potentially enhancing iron acquisition capacity. Two non-ST11 isolates (s26 and s29) exhibited an increased abundance of adhesin genes (*fimH*), suggesting a greater potential for host tissue colonization and invasion. In contrast, the ST307 isolate (s2) lacked rmpA/rmpA2 but carried an expanded *kpn* gene repertoire (n=7), implying an alternative virulence strategy. Virulence determinant distribution was closely correlated to specific ST-KL combinations, suggesting that virulence gene acquisition was strongly lineage-dependent during CRKP evolution. Furthermore, the type I fimbriae gene (*fim*) was markedly enriched in isolates s2 (KL02), s26 (KL101), s27 (KL8), and s29 (KL145) compared with those of KL25 and KL64 capsule types.

Resistance genes were identified in 29 isolates. The carbapenemase gene *blaKPC-2* was the predominant resistance determinant, detected in 23 isolates (79.31%). All ST11-KL25 isolates carried a broad spectrum of resistance genes, including *omp, pmr, qnr, ram, rmt, shv, sul, tem, tet*, and *tol*, whereas the ST307 isolate contained *oxa* and *oqx* genes (**Figure 2B**). Notably, the ST45-1LV isolate (s4) exhibited the highest level of antimicrobial susceptibility, accompanied by an absence of multiple resistance genes. These findings suggested that specific clone-capsule type combinations potentially promoted the establishment and dissemination of distinct resistance mechanisms, influencing both virulence potential and bacterial adaptability. KL25-type isolates displayed enhanced biofilm-forming ability, associated with a higher *rmp* gene prevalence, which potentially contributed to their persistence in clinical environments.

### 3.5 Phylogenetic analysis

**Figure 3:**
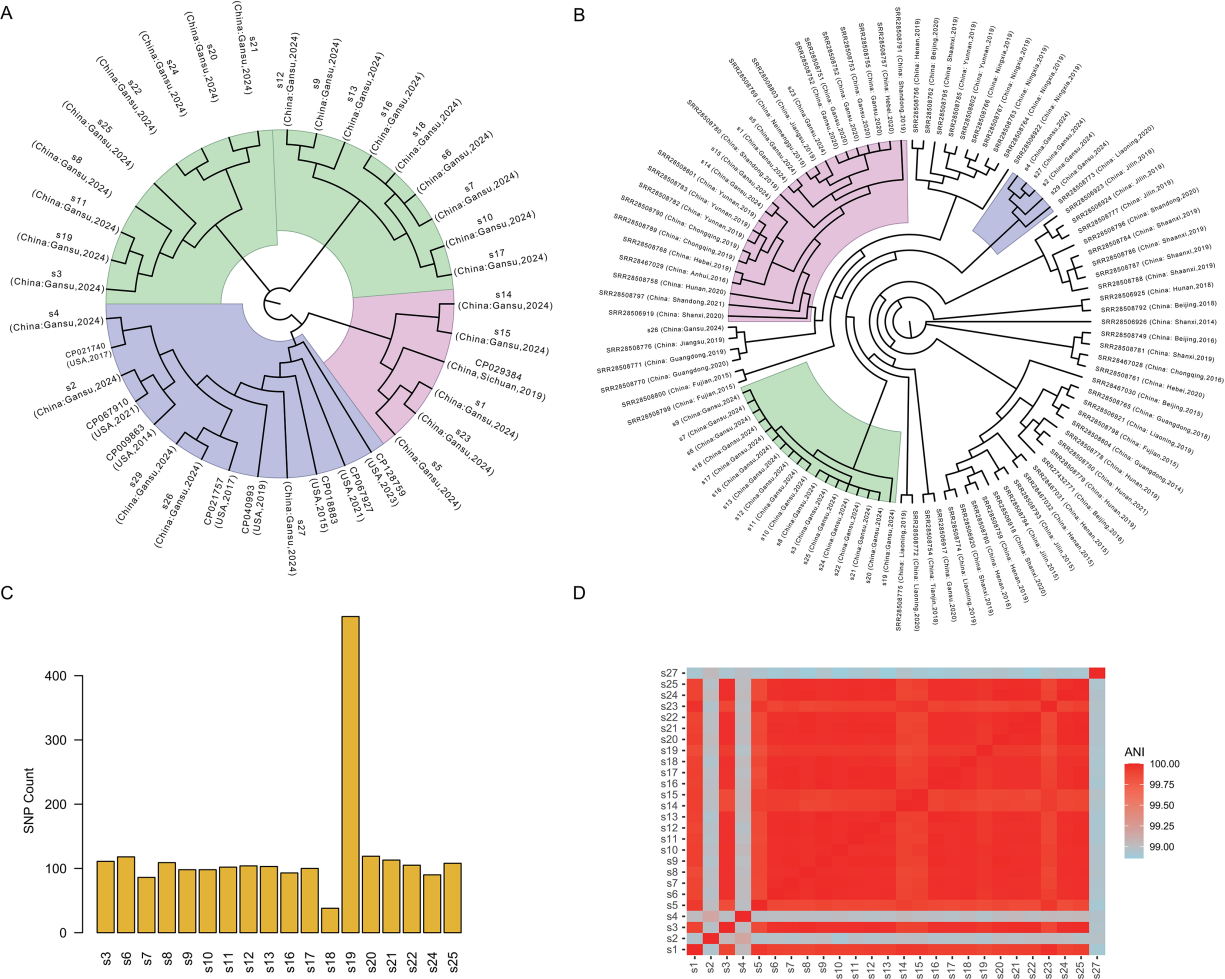
Phylogenetic analysis of carbapenem-resistant Klebsiella pneumoniae (CRKP) strains. **(A)** Whole-genome SNP-based phylogenetic tree illustrating evolutionary relationships with global reference isolates. **(B)** Regional phylogenetic context of study isolates compared to CRKP isolates from other regions in China. **(C)** Histogram depicting the distribution of pairwise SNP differences among ST11-KL25 isolates. **(D)** Heatmap of average nucleotide identity (ANI) values showing high genomic similarity among ST11 isolates.

Phylogenetic analysis demonstrated that the 28 local isolates clustered into three distinct lineages corresponding to their ST and capsular genotype (KL): ST11-KL25 (green), ST11-KL64 (pink), and other types (blue) (**Figure 3A**). When compared with globally representative *K. pneumoniae* genomes, ST11 clones identified here were closely related to high-risk lineages previously reported in China (Kang et al., 2024), but showed clear genetic divergence from epidemic clones prevalent in Europe and North America, such as ST258 (**Figure 3A**). A comparison with 72 K*. pneumoniae* strains from other regions in China revealed that the 18 ST11-KL25 isolates formed a strongly supported monophyletic cluster, suggesting a shared evolutionary origin. Similarly, ST11-KL64 isolates formed a distinct and well-defined clade (**Figure 3B**), encompassing strains from multiple geographical regions. Temporally, isolates collected before 2019–2020 exhibited greater genomic similarity, indicating relatively conserved mutation patterns in earlier CRKP populations.

Non-ST11 strains (s2, s4, s26, s27, and s29) formed independent clades, reflecting localized genetic divergence and possible region-specific evolution. The phylogenetic tree further revealed evolutionary linkages between CRKP isolates from Gansu and those circulating in other provinces in China. Several clones demonstrated interregional clustering-for instance, Shanxi, Inner Mongolia, and Ningxia isolates exhibited high genetic similarity to those from Gansu-suggesting potential cross-regional dissemination. ST11-KL25 isolate predominance was consistent with national epidemiological trends, highlighting the extensive spread and persistence of this high-risk clone in Chinese healthcare settings (Hu et al., 2024). Comparative SNP analysis of the 18 ST11-KL25 isolates showed limited genetic variation (range, 38-492; median, 103.5) (**Figure 3C**), indicating a possible nosocomial transmission. ANI analysis confirmed that all ST11 isolates shared>99.9% nucleotide identity (**Figure 3D**), supporting their derivation from a common ancestral lineage. Collectively, these findings indicated that the CRKP population in the studied hospitals was primarily driven by a small number of successful epidemic clones-particularly ST11-KL25 and ST11-KL64-that had adapted and persisted within local hospital environments.

### 3.6 Pan-genome analysis

**Figure 4:**
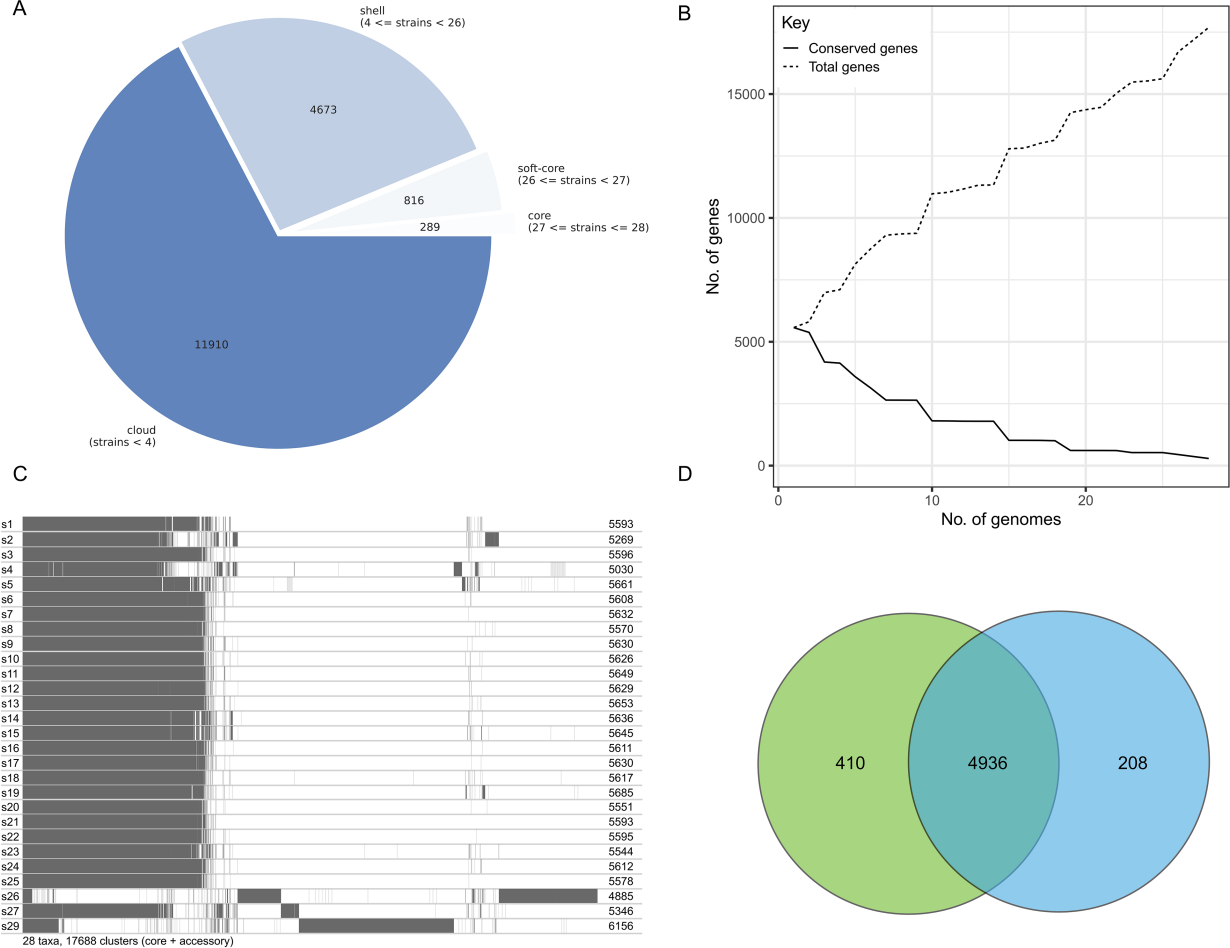
Pan-genome analysis of carbapenem-resistant Klebsiella pneumoniae (CRKP) isolates. **(A)** Pie chart depicting the composition of the pan-genome, showing the distribution of core, soft-core, shell, and unique gene fractions. **(B)** Core gene accumulation curve illustrating the trend as genome number increases, indicating saturation of the core gene set. **(C)** Bar plot displaying the number of strain-specific genes per isolate. **(D)** Venn diagram comparing the core genomes of ST11-KL25 and ST11-KL64 lineages, highlighting genomic differences between the two predominant clonal types.

Pan-genome analysis of the 28 high-quality CRKP genomes (two low-quality assemblies were excluded) identified 17,688 gene families (**Figure 4A**). The core and soft-core genome-defined as genes present in≥95% of isolates-comprised 1,105 genes, accounting for 6.28% of the total pan-genome. The shell genome (genes present in 15%-95% of isolates) contained 4,673 genes, whereas the accessory or unique genome (genes present in<15% of isolates) included 11,910 genes. The core gene accumulation curve plateaued after approximately 20 genomes (**Figure 4B**), indicating adequate sampling depth and a stable estimation of the CRKP core genome. Distinct genomic diversity was observed among non-ST11 isolates (s2, s4, s26, s27, and s29), which possessed the largest numbers of strain-specific genes and exhibited genomic composition markedly divergent from ST11-type isolates (**Figure 4C**). Focusing on the two predominant lineages-ST11-KL25 and ST11-KL64-a combined total of 6,626 gene families was identified, of which 4,936 genes (74.49%) were shared among all isolates, representing their core genome. When focusing on these two lineages separately, ST11-KL25 contained 5,346 core genes, whereas ST11-KL64 harbored 5,144 (**Figure 4D**).

**Figure 5:**
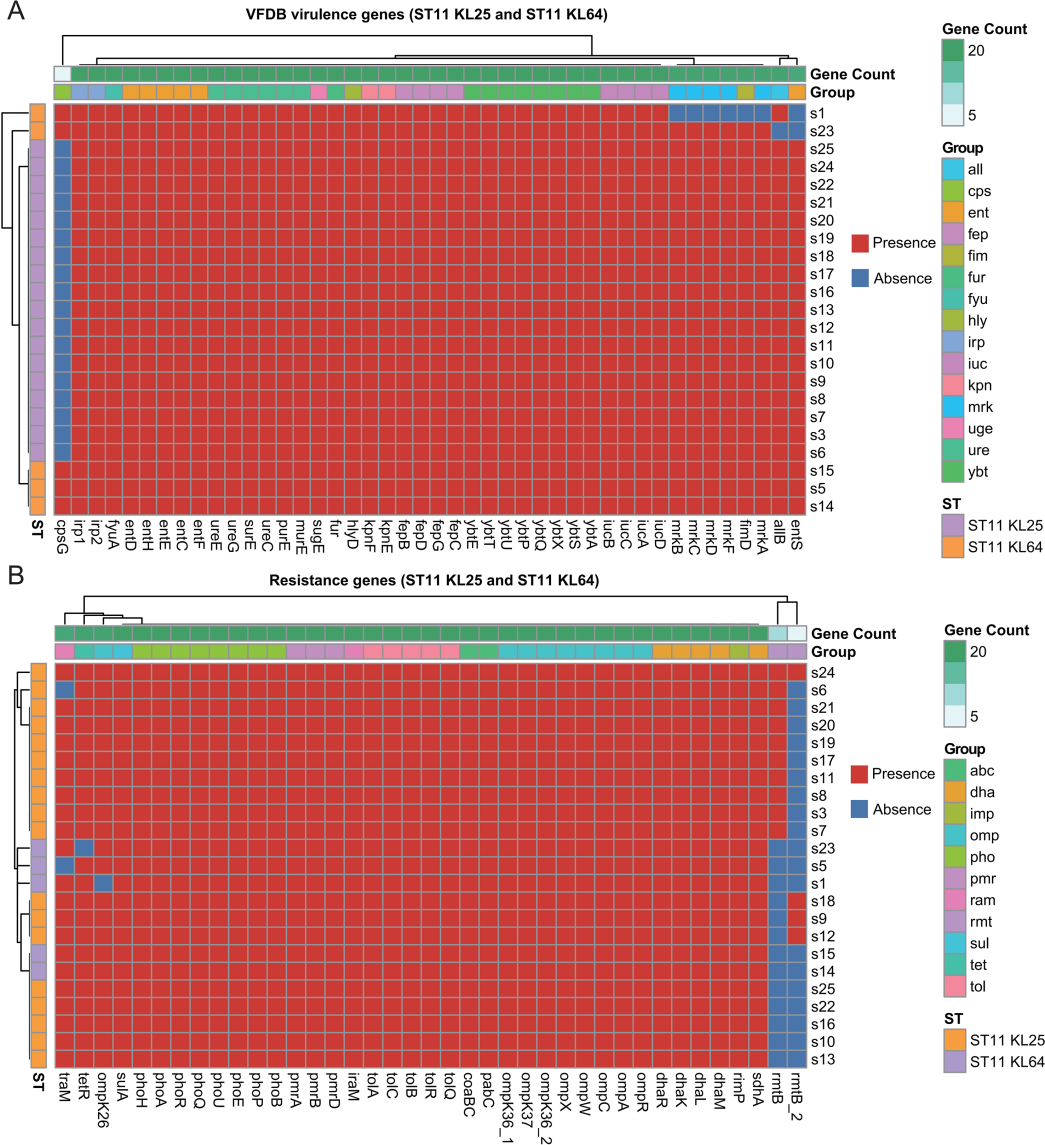
Virulence and antimicrobial resistance gene profiles in carbapenem-resistant Klebsiella pneumoniae (CRKP) isolates. **(A)** Heatmap displaying the distribution of virulence-associated genes across strains, highlighting lineage-specific patterns. **(B)** Heatmap illustrating the distribution of resistance genes, revealing a conserved core resistome within ST11 clones.

Further genomic characterization revealed that>90% of identified virulence-associated genes were conserved in the ST11 core genome (**Figure 5A**). Notably, the capsule biosynthesis gene *cpsG* (encoding phosphomannomutase CpsG) was absent in all KL25 isolates but present in all KL64 isolates. The *entS* gene (enterobactin transporter EntS) was missing in two KL64 isolates, and adhesion-associated genes-including one *fim* and five *mrk* variants-were absent in isolate s1. Resistance gene profiling demonstrated that most antimicrobial resistance genes were conserved in the ST11 core genome, regardless of KL subtype (**Figure 5B**), indicating strong genomic stability across these dominant lineages. However, *rmt* genes (*rmtB and rmtB_2*) were entirely absent in KL64 isolates but present in most KL25 isolates. These genomic patterns correlated with phenotypic susceptibility profiles: all KL25 isolates exhibited 100% resistance to amikacin, whereas 60% of KL64 isolates remained susceptible, consistent with previous findings (Wu et al., 2024).

Further analysis of the soft-core and unique gene repertoires of ST11-KL25 and ST11-KL64 lineages revealed notable functional distinctions. The KL25 lineage contained 355 soft-core genes (**Supplementary Table 5**), primarily associated with genomic recombination and transposition, horizontal gene transfer, antibiotic resistance, stress responses, and phage-related functions. In comparison, KL64 possessed 467 soft-core genes (**Supplementary Table 6**), enriched in similar biological processes, including genomic recombination and transposition, horizontal gene transfer, antibiotic resistance, energy production and metabolism, and phage-related activities. The KL25-specific gene set comprised 66 genes (**Supplementary Table 7**), predominantly involved in capsule and lipopolysaccharide (LPS) biosynthesis and transport, as well as horizontal gene transfer and phage-associated mechanisms. In contrast, the KL64-specific repertoire included 69 genes (**Supplementary Table 8**), mainly related to capsule and LPS biosynthesis, genetic regulation, horizontal gene transfer, and DNA maintenance and repair pathways.

### 3.7 Plasmid and mobile genetic element profiling

**Figure 6:**
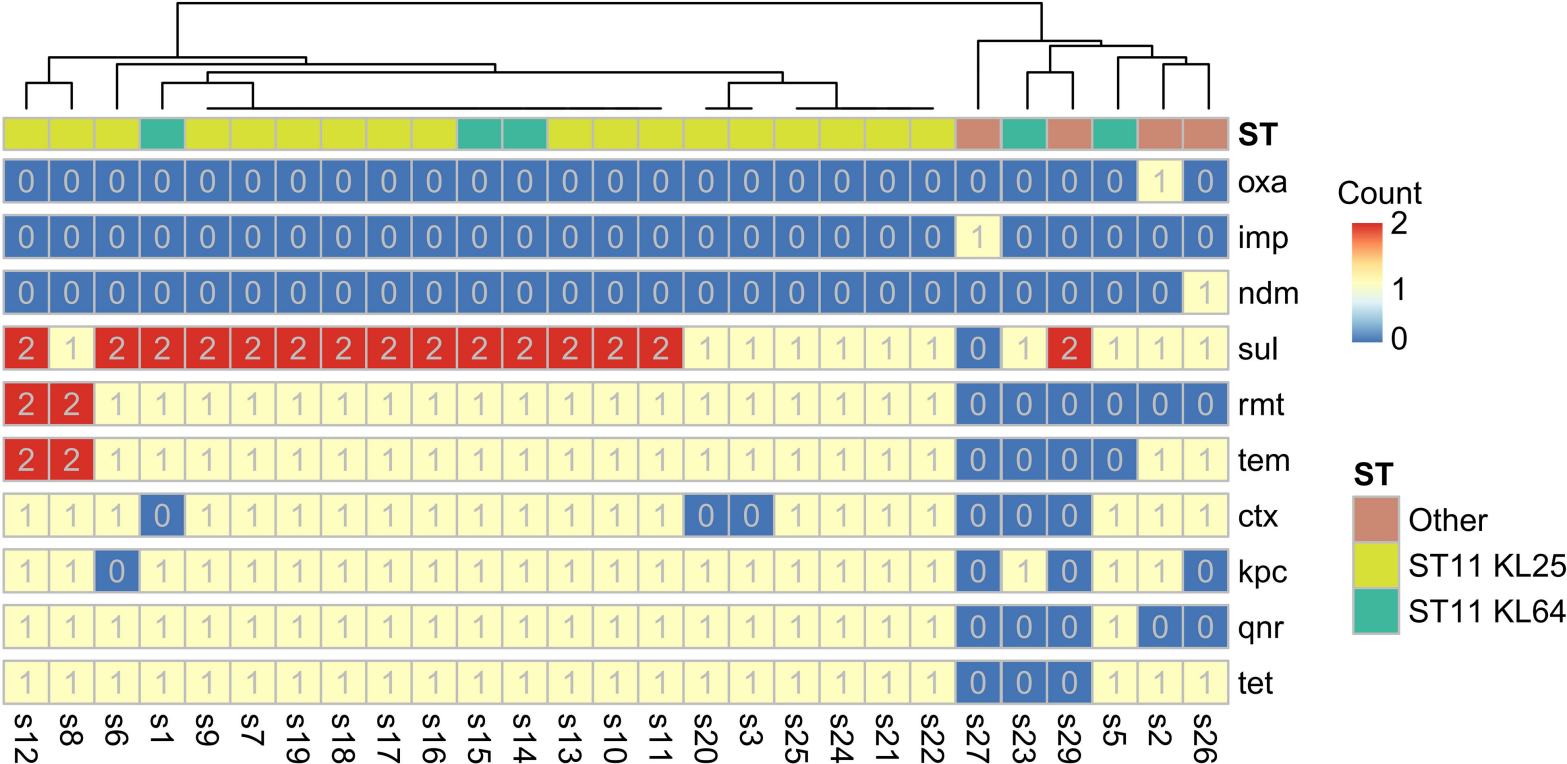
Heatmap analysis of resistance gene distribution across plasmids and chromosomes, revealing the genomic localization of major carbapenemase genes and other antimicrobial resistance determinants.

Plasmid analysis revealed that the 28 CRKP isolates harbored an average of 17.43 plasmids per strain, with sizes ranging from 5 kb to 212 kb. The majority of plasmids were of *Proteobacteria* origin (99.67%). Resistance gene distribution analysis demonstrated that the carbapenemase gene *blaKPC-2* was predominantly plasmid-borne. Furthermore, resistance genes including *ctx, imp, ndm, oxa, qnr, rmt, sul, tem*, and *tet* were also located on plasmids, whereas genes such as *pmr, shv, ram, omp*, and *tol* were chromosomally encoded (**Figure 6**). Prophage annotation identified 385 prophage regions across the 28 CRKP isolates, with an average of 5.14 complete and 8.61 incomplete prophages per strain (**Supplementary Table 9**). Prophage regions ranged in size from 3.1 kb to 58.6 kb and were predominantly associated with *Klebsiella* (46.94%), *Salmonella* (19.05%), and *Enterobacter* (17.69%) genera.

## 4 Discussion

CRKP has become a major global public health concern due to its extensive multidrug resistance and association with life-threatening infections, as characterized by high mortality rates (Antimicrobial Resistance Collaborators, 2022). The CPS, a key virulence determinant of *K. pneumoniae*, has pivotal roles not only in immune evasion but also in antimicrobial resistance evolution and persistence, and to date, at least 79 distinct capsular types-designated K types-have been identified, exhibiting substantial genetic and phenotypic diversity (Follador et al., 2016; Pan et al., 2015). A nationwide longitudinal retrospective study in China reported an evolving virulence landscape among CRKP strains, with KL47 and KL64 emerging as dominant capsular types. This study also revealed marked geographical heterogeneity in K-type distribution: KL64 predominated in East and Central China, whereas KL47 was more prevalent in the North and Northeast. Additionally, several region-specific K types, such as KL10 in Northeast China, displayed localized endemic patterns (Hu et al., 2024). According to 2024 CHINET surveillance data, *K. pneumoniae* accounted for 19.4% of Gram-negative bacterial isolates in China, with resistance rates continuing to rise-particularly against aminoglycosides (e.g., amikacin, 67.1%) and tigecycline (6.9%)-underscoring a growing epidemiological burden associated with CRKP (https://www.chinets.com/Data/GermYear).

In our study, WGS and comparative genomic analyses were performed on 28 clinical CRKP isolates from two tertiary hospitals in Gansu Province, western China. The findings revealed that ST11-CRKP strains carrying the KL25 capsular serotype were most prevalent, followed by KL64 and several less common types. The dominant clone, ST11-KL25, possessed numerous virulence and antimicrobial resistance genes, suggesting high pathogenic potential. Reports of this lineage remain limited, with the earliest documented cases originating from Henan Province (2020) and Shanxi Province (2024) (Fu et al., 2020; Wang et al., 2025). Notably, ST11-KL25 has been recognized as a typical “superbug”, characterized by the convergence of multidrug resistance and hypervirulence, thereby necessitating strengthened surveillance and infection control efforts. Recent studies conducted (between 2024 and 2025) reported that ST11-KL25 hypervirulent CRKP (hv-CRKP) isolates in China exhibited remarkable genomic homogeneity and often acquired virulence plasmids-such as pVir harboring rmpA2 and iucABCD-to mediate the fusion of resistance and virulence traits, thus facilitating rapid clonal dissemination (Kang et al., 2024). In a 2024 investigation at a tertiary hospital in Sichuan Province (Western China), ST11-KL64 carbapenem-resistant hypervirulent *K. pneumoniae* (CR-HvKP) was shown to be the predominant cause of nosocomial infections, accounting for 52.9% of CRKP isolates. This lineage was significantly associated with intensive care unit (ICU) admissions (odds ratio [OR]=2.939, p=0.056) and prior carbapenem exposure (OR = 2.718, p=0.071) (Yang et al., 2025). These trends were consistent with observations from Gansu Province, indicating active regional clonal transmission and ongoing ST11-KL25 and ST11-KL64 adaption in hospital environments.

WGS revealed that our CRKP isolates possessed relatively large genomes (average, 5.65 Mb) and contained numerous mobile genetic elements and plasmids. Plasmid profiling demonstrated that key resistance genes, including *blaKPC-2*, were primarily located on transferable plasmids, thereby potentially enhancing the dissemination of horizontal carbapenem resistance. These genomic characteristics potentially facilitated the co-transfer of antimicrobial resistance determinants and virulence factors, providing a molecular basis for rapid CRKP spread and environmental adaptability in healthcare settings. A recent nationwide genomic survey (2025) comprising 5,036 *K. pneumoniae* isolates reported that CR-hvKP accounted for 44.6% of all CRKP strains, with *blaKPC* detected in 92.1% of isolates, and meanwhile, the prevalence of *blaNDM* and *blaOXA-48*-like genes has continued to increase (Li L. et al., 2025). Notably, high-virulence determinants such as aerobactin (iuc locus) and yersiniabactin (ybt locus) were shown to coexist in 93.6% of isolates (Li X. et al., 2025). In a regional investigation (2018-2022) in eastern Sichuan*, K. pneumoniae* ST11 isolates producing KPC-2 accounted for 74.2% of CRKP cases, whereas NDM-5-producing strains displayed diverse sequence types (e.g., ST29 and ST2407) and frequently carried conserved IncX3 plasmids that promoted efficient horizontal gene transfer (Yuan et al., 2024). Collectively, these findings support our resistance gene profiles in ST11-KL25 and ST11-KL64 lineages. The emergence and dissemination of carbapenem-resistant hypervirulent (CR-hv) plasmids-capable of co-harboring both resistance and virulence determinants-has become increasingly common across China (Tian et al., 2022).

Recombination events in the CPS(a key virulence determinant of *K. pneumoniae*) biosynthetic gene cluster have pivotal roles promoting genetic diversity and facilitating the acquisition of novel antimicrobial resistance mechanisms (Guo et al., 2022). Accumulating evidence now indicates that specific capsular serotypes are strongly correlated with CRKP transmissibility and outbreak potential, thereby contributing to rapid clinical progression and adverse outcomes. Accordingly, the detailed characterization of K-type distribution is essential for effective epidemiological surveillance and infection control efforts (Guo et al., 2022). Simultaneously, monitoring regional ST dynamics can provide critical insights into strain variation and population structures across geographical regions, thereby informing targe ted monitoring and intervention strategies (Luo et al., 2023). Previous investigations have documented a marked subclonal shift in the predominant ST11-CRKP lineage-from the formerly dominant KL47 serotype to the recently emergent KL64-suggesting that ST11-KL64 possesses enhanced adaptability compared to its KL47 counterpart (Zhou et al., 2020). In our study, most CRKP isolates belonged to the ST11 lineage, with KL25 the predominant capsular serotype. The ST11-KL25 genotype exhibited the highest detection frequency. Importantly, these ST11-KL25 isolates not only carried the carbapenemase gene *blaKPC-2*, but also co-harbored virulence-associated genes such as *rmpA/rmpA2* and iron acquisition determinants, indicating the evolutionary convergence of high-level antimicrobial resistance and augmented virulence potential in this emerging clone.

Phylogenetic analysis revealed a high degree of genetic homogeneity among ST11 strains from this region, with a median SNP difference of only 103.5, suggesting potential nosocomial transmission. Pan-genome analysis indicated that core genes shared between ST11 and non-ST11 sequence types accounted for only 6.28% of the total pan-genome, reflecting substantial genomic plasticity across different capsular serotypes. This genomic flexibility likely represented a key mechanism underlying adaptive CRKP evolution in diverse ecological and clinical environments. A previous population structure study using clustering analysis suggested that KL25 strains may have evolved from KL64 lineages (Wang et al., 2025). Our findings support this hypothesis, demonstrating that ST11-KL25 and ST11-KL64 isolates shared highly conserved core genomes but differed in distinct virulence and resistance determinants. Notably, *rmt* gene family members were prevalent in KL25 strains but absent in KL64, a genetic feature consistent with observed differences in amikacin susceptibility between the two lineages. A recent 2025 investigation confirmed that the hypervirulent phenotype of ST11-KL64 isolates in western China (e.g., Sichuan) was strongly associated with the universal presence (100%) of the *iucA* gene. Moreover, the emergence of a novel wzi752 allele-phylogenetically related to KL47-suggested ongoing evolutionary diversification within the ST11 lineage (Li L. et al., 2025). In parallel, microbiome studies have shown that the intestinal microbiota confers protective effects against CRKP colonization; however, the disruption of this microbial barrier in ICU may facilitate pathogen persistence and transmission (Yang et al., 2025).

Although non-ST11 strains were less prevalent in our study, they exhibited distinct genomic characteristics and unique virulence gene distributions, potentially representing emerging epidemic lineages. Among these, the ST307 isolate harbored a specific *kpn* gene cluster, suggesting a divergent pathogenic mechanism that warrants further investigation. A recent global study of 95 ST307 Klebsiella pneumoniae genomes found that 93.7% (89/95) harbored the blaCTX-M-15 gene (Wyres et al., 2019), which is associated not only with extended-spectrum β-lactam resistance but also with key virulence determinants-including hypermucoviscosity and enhanced siderophore-mediated iron acquisition. Moreover, the majority of these ST307 isolates share the KL102 K-antigen locus (Peirano et al., 2020), a finding concordant with our own genomic characterization. Critically, ST307 is increasingly linked to severe clinical outcomes-including bloodstream infections and sepsis-and exhibits multidrug resistance that severely constrains therapeutic options. These features collectively underscore the urgent need for enhanced genomic surveillance and rigorous, evidence-based infection prevention and control strategies to curb its dissemination and mitigate its growing public health burden (Sheng et al., 2025). Our findings also provide valuable genomic evidence informing CRKP prevention and control strategies. Collectively, our data indicate that CRKP dissemination in hospital environments is predominantly driven by a limited number of successful epidemic clones-particularly ST11-KL25 and ST11-KL64-which have acquired multiple antimicrobial resistance and virulence determinants conferring strong adaptive advantages. Future research should focus on elucidating the evolutionary dynamics and transmission pathways of these high-risk clones, emphasizing a need for targeted interventions such as reinforced infection control measures and the development of novel antimicrobial therapies. Limiting horizontal gene transfer and curbing mobile genetic element spread are critical components of effective CRKP containment approaches. A 2025 longitudinal study at a hospital in Lishui, Zhejiang Province, reported increased CRKP detection rates, from 9.77% in 2015 to 10.38% in 2024, which were largely driven by sustained clonal expansion of the ST11 *blaKPC-2* lineage, underscoring the importance of intestinal colonization surveillance and proactive infection control (Liu et al., 2025). At the national level, clustered CR-hvKP transmission has been documented, with 89.0% of isolates grouped into 131 clonal clusters and primarily concentrated in eastern China. Nevertheless, increasing regional connectivity has also been observed in western provinces such as Sichuan (Li X. et al., 2025), highlighting a requirement for enhanced cross-regional surveillance networks and coordinated public health responses.

This study has several limitations. The relatively small sample size and inclusion of isolates from only two healthcare institutions may limit the generalizability of these findings across Gansu Province. Broader, multicenter surveillance efforts incorporating regionally representative datasets and detailed clinical outcome information are required to achieve a more comprehensive understanding of CRKP epidemiology, risk factors, and treatment responses. Laboratory-based investigations should further explore the biological characteristics of dominant clones, including biofilm formation, virulence phenotypes, and underlying resistance mechanisms. Sustained genomic surveillance programs are also essential for tracking the evolutionary dynamics of circulating CRKP strains and anticipating novel clone emergence. Integrative approaches that combine genomic data with clinical metadata may facilitate the development of predictive CRKP infection risk models in guiding individualized therapeutic decision-making.

Moreover, given the potential for pediatric intestinal colonization by CRKP, the early implementation of molecular screening and antimicrobial stewardship programs in western China is strongly recommended to mitigate the escalating threat posed by hv-CRKP.

In conclusion, we used WGS and comparative genomic analyses to elucidate the molecular characteristics and epidemiological trends underpinning CRKP in Gansu Province. Our findings underscore the importance of continuous capsular serotype surveillance to inform future vaccine development and targeted immunoprophylaxis against K. pneumoniae. The ST11-KL25 clone strain dominates in terms of carrying multiple antimicrobial drug resistance and virulence determinants. Active hospital infection control measures, such as environmental disinfection, patient isolation, and antibacterial management programs, are necessary to curb its spread. Additionally, more effective antibacterial drugs should be continuously developed to address infections, and they should be used rationally to avoid the emergence of drug-resistant strains.

## Data Availability

The datasets presented in this study can be found in online repositories. The names of the repository/repositories and accession number(s) can be found in the article/**Supplementary Material**.
